# Effects of Organ System Courses of the First Two Years of Medical School on the Performance of the Comprehensive Osteopathic Medical Licensing Examination of the United States (COMLEX-USA) Level 2-Cognitive Evaluation

**DOI:** 10.7759/cureus.25939

**Published:** 2022-06-14

**Authors:** Kevin McNeil, Vahid Mashhouri, Payton Christensen, Han Wang, Qing Zhong

**Affiliations:** 1 Medical School, Rocky Vista University College of Osteopathic Medicine, Ivins, USA; 2 Medical School, Rocky Vista University College of Osteopathic Medicine, Parker, USA; 3 Department of Data Analytics, Shanghai CraiditX Technologies Ltd., Shenzhen, CHN; 4 Department of Bioscience, Rocky Vista University College of Osteopathic Medicine, Ivins, USA

**Keywords:** third semester, principles of clinical medicine (pcm), cardiovascular system (cv), comprehensive osteopathic medical licensing examination of the united states level 1 (comlex level-1), comprehensive osteopathic medical licensing examination of the united states level 2-cognitive evaluation (comlex level 2-ce)

## Abstract

Objectives: The Comprehensive Osteopathic Medical Licensing Examination of the United States (COMLEX-USA) Level 2-Cognitive Evaluation (CE) is a board examination that medical students usually take in the third or fourth year of medical school. A few researchers have investigated the prediction of COMLEX Level 2-CE scores based on the performance in third-year clerkships. However, given how close the clerkships are to the board exam, this type of prediction is too late for students to have adequate time to get assistance to prepare for COMLEX Level 2-CE. We aimed to investigate the predictive value of each organ system course during the first two years in predicting COMLEX Level 2-CE performance. Our findings will help students at risk focus on important basic and clinical sciences much earlier before preparing for COMLEX Level 2-CE.

Methods: Academic data from students enrolled at Rocky Vista University College of Osteopathic Medicine from 2011 to 2017 were retrieved. Data included the Medical College Admission Test (MCAT) scores, course grades in the first two years of medical school, COMLEX Level 1 scores, and COMLEX Level 2-CE scores. Pearson correlation coefficients, a multiple linear regression model, and a backward stepwise regression model were generated for analysis.

Results: The highest correlation with COMLEX Level 2-CE scores was the COMLEX Level 1 score, followed by the performances in the third-semester Cardiovascular System II (CV II) and Renal System II (REN II) courses. Multiple linear regression and backward stepwise regression predictive models found that scores on third-semester CV II and Principles of Clinical Medicine III (PCM III) were the most significant predictors of performance on Level 2-CE. Both models explained 46% of the variance in COMLEX Level 2-CE scores.

Conclusions: Performances in third-semester courses are the most important predictors of COMLEX Level 2-CE scores.

## Introduction

Students in osteopathic medical schools must pass the Comprehensive Osteopathic Medical Licensing Examination of the United States (COMLEX-USA) series Level 1 and Level 2-Cognitive Evaluation (CE) to graduate and match to residency programs. These examinations are equivalent to the United States Medical Licensing Examination (USMLE) Step 1 and Step 2 Clinical Knowledge (CK) tests in allopathic medicine, respectively [1]. Students usually take COMLEX Level 2-CE in the third or fourth year of medical school. All board examinations, including the COMLEX series and USMLE series, are important in the residency application. Higher scores on COMLEX Level 2-CE are associated with an increased likelihood of matching to first choices of residency programs and more competitive specialties [2]. Furthermore, COMLEX Level 2-CE scores are more crucial than ever for residency placement since reporting “pass or fail only” has been applied to USMLE Step 1 and COMLEX Level 1 on and after January 26, 2022, and May 10, 2022, respectively [3,4]. Therefore, early prediction of performance on COMLEX Level 2-CE is critical, so that students who are at risk of scoring poorly will have enough time to get assistance and perform better on this exam.

Although some studies have explored the prediction of performance on COMLEX Level 1 [1,5], only a few studies have been focused on the prediction of COMLEX Level 2-CE performance [6-8]. Preadmission undergraduate science grade point average (sciGPA) or Medical College Admission Test (MCAT) scores show a small but significant correlation with COMLEX Level 2-CE performance [6-8]. Academic performance during the first two years of medical school has a stronger correlation with COMLEX Level 2-CE scores as compared to preadmission sciGPA and MCAT scores [7]. In addition, Evans et al. investigated academic data from 1,254 students from 12 osteopathic medical schools. They found that the highest correlation with the COMLEX Level 2-CE score was the COMLEX Level 1 score, followed by the performance in the first two years of medical school, total grade point average (GPA) as a fourth-year student, and clinical GPA [9]. Furthermore, since the Comprehensive Osteopathic Medical Achievement Test (COMAT) series of subject examinations was installed in 2011, two studies indicated that scores on COMAT explained 63-68% of the variance in Level 2-CE scores, with COMAT Internal Medicine and Emergency Medicine displaying the highest correlations [10,11]. Using the COMAT performance to predict COMLEX Level 2-CE is too late for students since they will not have enough time to modify and improve their preparation for COMLEX Level 2-CE.

There is no research investigating the impact of each organ system course during the preclinical years on the performance of COMLEX Level 2-CE. Our goal is to find the earliest predictors of COMLEX Level 2-CE scores in the preclinical years so that students can focus on improving earlier.

## Materials and methods

Data

As described in our previous publication [1], Rocky Vista University College of Osteopathic Medicine (RVUCOM) has a stepwise organ system-based curriculum for the first two preclinical years. Each organ system is covered twice, with the first course in the first year focusing on anatomy and physiology, and the second course in the second year focusing on pathology, pharmacology, and diagnosis and treatment. Details of the major courses in the first three semesters are listed in our previous study [1].

Participants

Academic data of students who enrolled at RVUCOM between 2011 and 2017 were retrieved. The project entitled “Using Simulation Modeling to Predict Failure on COMLEX 1 and 2 at First Attempt Through a Longitudinal Investigation” was approved by the IRB committee of RVUCOM (IRB number: IRB #2019-0079) [1]. Informed consent was waived because the IRB committee determined that this project was exempt. De-identified data were disclosed to the investigators.

Independent variables

The MCAT scores, final grades in each course in the first two years of RVUCOM, and COMLEX Level 1 scores at the first attempt were used. As described in our previous publication [1], if students took the MCAT more than once, average scores across all MCAT attempts were calculated. The average scores in all courses of the first year, all courses of the second year, and all preclinical courses in the first two years of medical school were calculated and were used as independent variables as well.

Dependent variable

Scores on COMLEX Level 2-CE from 586 students on their first attempt were collected.

Correlations between independent variables and scores of COMLEX Level 2-CE

Pearson correlation coefficients (R) were used to measure the correlations between independent variables (MCAT scores, preclinical course grades, the average scores in all courses of the first year, second year, and first two years, and COMLEX Level 1 scores) and the dependent variable (COMLEX Level 2-CE scores).

Multiple linear regression model

A multiple linear regression model was generated. This predictive model is based on academic performances in organ system courses of preclinical years. The independent variables (27 in total) included all final grades in each course in the first two years of medical school and did not include MCAT and COMLEX Level 1 scores. The dependent variable was the score on COMLEX Level 2-CE. The final formula consisted of 27 variables.

Backward stepwise regression model

Independent variables included scores in each course in the first two years and did not include MCAT and COMLEX Level 1 scores. The dependent variable was the score on COMLEX Level 2-CE. In the final formula, only significant variables have remained because insignificant independent variables were deleted sequentially.

Statistical analysis

All analyses were done using either IBM SPSS version 20 (IBM Corp., Armonk, NY) or SigmaPlot 14 (Systat Software Inc., San Jose, CA).

## Results

The correlations between MCAT scores, the average scores in the first two years, and performances in board examinations

A total of 906 students finished COMLEX Level 1, and 586 students took COMLEX Level 2-CE. As shown in Table 1, MCAT scores and performances in the first two years were significantly correlated with COMLEX Level 1 scores and COMLEX Level 2 scores, respectively. The highest correlation with the COMLEX Level 1 score was the average score in second-year courses (r = 0.76), followed by the average score in the first two years’ courses (r = 0.73) and the average score in first-year courses (r = 0.66). The highest correlation with performance on COMLEX Level 2-CE was the COMLEX Level 1 score (r = 0.76), followed by the average score in second-year courses (r = 0.71), the average score of all the first two years’ courses (r = 0.66), and the average score in first-year courses (r = 0.59). MCAT scores were weakly correlated with the COMLEX Level 1 score (r = 0.18) and COMLEX Level 2-CE performance (r = 0.14), respectively.

**Table 1:** Correlation between MCAT score, first two years’ performances, and performances in board examinations *** p < 0.00001; ** p < 0.001. MCAT = Medical College Admission Test; COMLEX Level 1 = Comprehensive Osteopathic Medical Licensing Examination of the United States Level 1; COMLEX Level 2-CE = Comprehensive Osteopathic Medical Licensing Examination of the United States Level 2-Cognitive Evaluation; First-year average score = the average score of all first-year courses; Second-year average score = the average score of all second-year courses; First two years’ average score = the average score of all first two years’ courses.

Pearson coefficient (R)	COMLEX Level 1 (N = 906)	COMLEX Level 2-CE (N = 586)	P
MCAT	0.18	0.14	**
First-year average score	0.66	0.59	***
Second-year average score	0.76	0.71	***
First two years’ average score	0.73	0.66	***
COMLEX Level 1	-	0.76	***

The correlations between performance in each course and COMLEX Level 2-CE scores

Since we found that performance throughout the first two years was highly correlated with COMLEX Level 2-CE, we further investigated the effects of each organ system course on COMLEX Level 2-CE performance. Table 2 shows the correlations between the scores in each course with performance on COMLEX Level 2-CE in descending order. From Table 2, performance in all courses was significantly correlated with COMLEX Level 2-CE scores, with Pearson R ranging from 0.33 to 0.65. The two courses with the highest correlations were second-year third-semester Cardiovascular System II (CV II) and Renal System II (REN II), with a correlation of 0.65 and 0.64, respectively. Thus, both CV II and REN II scores independently explain approximately 41% of the variance in COMLEX Level 2-CE scores. The top 12 courses were all second-year courses, except the 10th, which was first-year second-semester Endocrine/Reproductive System I. Compared to second-year courses, first-year courses had lower correlations with COMLEX Level 2-CE scores.

**Table 2:** Correlations between performance on each organ system course of first two years and scores of COMLEX Level 2-CE (N = 586) *** p < 0.00001.

Semester	Year	Course		Pearson R to COMLEX Level 2-CE	P
3^rd^	2	Cardiovascular System II	CV II	0.65	***
3^rd^	2	Renal System II	REN II	0.64	***
4^th^	2	Endocrine System II	ENDO II	0.62	***
4^th^	2	Neuroscience System II	NEURO II	0.60	***
3^rd^	2	Hematology/Lymphatic System II	HEME II	0.59	***
3^rd^	2	Gastrointestinal System II	GI II	0.58	***
4^th^	2	Musculoskeletal System II	MSK II	0.57	***
3^rd^	2	Respiratory System II	RESP II	0.57	***
4^th^	2	Reproductive System II	REPRO II	0.54	***
2^nd^	1	Endocrine/Reproductive System I	ENDO I	0.53	***
3^rd^	2	Principles of Clinical Medicine III	PCM III	0.52	***
4^th^	2	Psychiatry System	PSYCH II	0.51	***
2^nd^	1	Neuroscience System I	NEURO I	0.51	***
4^th^	2	Osteopathic Principle/Practice IV	OPP IV	0.50	***
1^st^	1	Cardiovascular System I	CV I	0.49	***
1^st^	1	Musculoskeletal System I	MSK I	0.47	***
4^th^	2	Principles of Clinical Medicine IV	PCM IV	0.47	***
1^st^	1	Renal System I	REN I	0.46	***
2^nd^	1	Gastrointestinal System I	GI I	0.45	***
3^rd^	2	Osteopathic Principle/Practice III	OPP III	0.45	***
2^nd^	1	Principles of Clinical Medicine II	PCM II	0.44	***
1^st^	1	Principles of Clinical Medicine I	PCM I	0.42	***
2^nd^	1	Osteopathic Principle/Practice II	OPP II	0.42	***
1^st^	1	Respiratory System I	RESP I	0.41	***
1^st^	1	Molecular Cellular System	MCM	0.40	***
1^st^	1	Hematology/Lymphatic System I	HEME I	0.36	***
1^st^	1	Osteopathic Principle/Practice I	OPP I	0.33	***

Multiple linear regression model

Although the multiple linear regression predictive model had 27 independent variables in the formula, only two variables were significant predictors of performance on COMLEX Level 2-CE, as shown in Table 3. Those two predictors were performances in two of the third-semester courses: CV II and Principles of Clinical Medicine III (PCM III). This model had a Pearson R of 0.70, and an adjusted R^2^ of 0.46, explaining 46% of the variance in COMLEX Level 2-CE scores.

**Table 3:** Regression models predicting COMLEX Level 2-CE scores CV II = Cardiovascular System II; PCM III = Principles of Clinical Medicine III; REN II = Renal System II; NS II = Neuroscience System II; MCM = Molecular Cellular System.

	Multiple linear regression		Backward stepwise regression	
	Coefficient	P	Coefficient	P
CV II	0.260	0.024	0.367	<0.001
PCM III	0.296	0.011	0.364	<0.001
REN II		>0.05	0.292	0.005
NS II		>0.05	0.350	<0.001
MCM		>0.05	0.186	0.011
All other courses	Included in the formula		Removed from the formula	
Adjusted R^2^	0.46		0.46	

Backward stepwise regression model

From the backward stepwise regression predictive model, there were only five variables that significantly predicted COMLEX Level 2-CE in the final formula, as shown in Table 3. Those variables were scores on the third-semester courses, including CV II, PCM III, and REN II, the fourth-semester Neuroscience System II (NEURO II) course, and the first-semester Molecular Cellular System (MCM) course. All other variables were found to be statistically insignificant and were removed from the formula sequentially. This model had a Pearson R of 0.68 and an adjusted R^2^ of 0.46. This model explained 46% of the variance in COMLEX Level 2-CE performance.

## Discussion

From our study, the highest correlation with COMLEX Level 2-CE performance was COMLEX Level 1 scores, followed by average scores in second-year courses. Our predictive regression models showed CV II and PCM III scores were the most significant predictors. Both models explained approximately 46% of the variance in the COMLEX Level 2-CE scores.

Our current study found that COMLEX Level 1 scores had the highest correlation with COMLEX Level 2-CE scores, with an R-value of 0.76. This was consistent with a 0.76 correlation reported by Evans et al. [9]. Therefore, it is reasonable to infer that a student who performs poorly on COMLEX Level 1 is at risk for poor performance on COMLEX Level 2-CE.

We found that third-semester and fourth-semester courses had higher correlations with performance on COMLEX Level 2-CE as compared to first-semester and second-semester courses. The highest correlation was the third-semester CV II course. Our two regression models provided convincing evidence that performance on CV II was the most important predictor. The backward stepwise regression model demonstrated that additional third-semester courses REN II and PCM III, fourth-semester NEURO II, and first-semester MCM were significant predictors of COMLEX Level 2-CE performance as well. In addition, our previous predictive models for COMLEX Level 1 have found that third-semester courses were most essential for predicting the COMLEX Level 1 score [1]. Since there is a high correlation between COMLEX Level 1 and Level 2-CE, it is not a surprise that third-semester courses are also strong predictors of COMLEX Level 2-CE scores. However, no explanation exists in the literature as to why these courses are so pivotal to Level 2-CE. The authors hypothesize that CV II and REN II courses involve physiology, pathology, and pharmacology, all of which contribute to building up the capacities to understand and correctly diagnose diseases. Those capacities are assessed in Level 2-CE, which evaluates the application and integration of knowledge between foundational biomedical sciences and clinical medicine. Students who perform poorly in third-semester courses, especially CV II, may need early intervention to prepare for COMLEX Level 2-CE.

Furthermore, another third-semester course (PCM III) was deemed an essential predictor according to both regression models. This is because PCM III primarily teaches internal medicine, emphasizing the diagnosis and treatment of diseases. It parallels the finding that the National Board of Osteopathic Medical Examiners (NBOME) COMAT Internal Medicine and Emergency Medicine performances in the third-year rotation are the most significant predictors for COMLEX Level 2-CE because Level 2-CE emphasizes primary care as a generalist examination [10,11]. If students perform poorly in PCM III, they may need additional assistance when preparing for Level 2-CE.

To our knowledge, our current study is the first to investigate the effect of performance in each preclinical organ system course on the COMLEX Level 2-CE scores. Our regression models provide early prediction of COMLEX Level 2-CE scores. Our study has the advantage of predicting COMLEX Level 2-CE performance one to two years in advance compared to predictive models based on COMAT performance. Thus, students who perform poorly in CV II and PCM III in the third semester will have enough time to get assistance and ultimately improve their performance on COMLEX Level 2-CE.

Limitations

Although our regression models explain 46% of the variance in COMLEX Level 2-CE scores, there is still 54% of the variance that cannot be attributed to performance during the first two years, which could be attributed to performance in the last two years of clinical rotations. We did not include performance on clinical rotations, clerkships, or clinical subject examinations, as we aimed to generate predictive models based on preclinical academic performance. Our study is based on academic data from one single institution and may not necessarily apply to other institutions with different curricula. In addition, some medical schools have changed to a pass/fail grading system, and our findings may not be as valuable for a medical school with this new grading system.

## Conclusions

There are significant correlations between COMLEX Level 1 scores, performance in the first two years of medical school, and COMLEX Level 2-CE scores. Among all courses, third-semester courses, especially CV II and PCM III, are the key predictors according to our regression models. Our regression models based on the performances during the first two years explain around 46% of the variance in COMLEX Level 2-CE scores. Our findings provide models for the earliest prediction of COMLEX Level 2-CE scores. At the end of the third semester, students who perform poorly in third-semester courses should receive assistance and intervention to better prepare them for COMLEX Level 2-CE that they must take one to two years later. In the future, investigating the effects of the third-semester courses on clinical subject performances may be necessary.
